# Bacterial community improves the volatile components coupled with abiotic factors during the spontaneous fermentation of Chinese strong-aroma *Baijiu*

**DOI:** 10.1016/j.fochx.2024.102068

**Published:** 2024-12-09

**Authors:** Bin Lin, Jie Tang, Qun Li, Liping Zhu, Wei Jiang, Hanbing Ke, Zhang Wen, Huaichen Liu, Shengzhi Yang, Qiang Yang, Shenxi Chen, Peijie Han

**Affiliations:** a Hubei Provincial Key Lab for Quality and Safety of Traditional Chinese Medicine Health Food, Jing Brand Research Institute, Jing Brand Co., Ltd., Daye 435100, PR China; b State Key Laboratory of Mycology, Institute of Microbiology, Chinese Academy of Sciences, Beijing 100101, PR China; c College of Life Science, University of Chinese Academy of Sciences, Beijing 100049, PR China

**Keywords:** Chinese *Baijiu*, Microbial succession, Aroma component, Physicochemical property, Functional profile

## Abstract

Flavor components largely depend on microbial activity and environmental conditions during traditional fermented food production. However, the microbial and abiotic contributions to the flavor of Chinese strong-aroma *Baijiu* (SAB) remain poorly understood. In this study, the composition and functional profiles of the fungal and bacterial communities changed significantly after fourteen days of grain fermentation. *Acetilactobacillus jinshanensis*, *Issatchenkia orientalis* and *Kazachstani humilis* became the dominant species as fermentation proceeded. Pathways related to lipid, protein and secondary metabolite metabolism were enriched during the middle and later stages of grain fermentation. Ethyl caproate, hexyl caproate, and ethyl lactate were identified as the main volatile components in fermented grain. *Lactobacillus* species were significantly related to volatile components. Compared with fungi, bacterial diversity accounted for 96 % of the variation in volatile components coupled with temperature, acidity and reducing sugar. This work provides insights into the production optimization and flavor enhancement of SAB by optimizing abiotic factors and microbial compositions.

## Highlights

• Effects of microbiota and abiotic factors on volatile were comprehensively studied.
• A total of 68 volatile components were detected in fermented grains.
• Microbiota and volatile components showed stage-special variation.
• Bacteria improve flavor formation coupled with temperature, acid and reducing sugar.
• *Lactobacillus* species were significantly related with volatile components.

## 1 Introduction

As the major fermented distilled beverage, *Baijiu* is widely popular among Chinese people around the world because of its unique flavor and taste. Chinese *Baijiu* can be divided into three basic aroma categories according to its flavor components and production technology: sauce-aroma, strong-aroma, and light-aroma (Jin et al., 2017). Among these types, strong-aroma *Baijiu* (SAB) makes up more than 50 % of the market share in China and plays a vital role in the Chinese food economy (Xu et al., 2023). Compared with the other types of *Baijiu*, the SAB fermentation process occurs in an underground pit cellar, in which raw grains are decomposed to generate new flavor substances (acids, alcohols, esters, etc.) by functional microbes (Xu et al., 2017). The fermentation of SAB is a spontaneous fermentation process with a multispecies microbial community in a pit cellar for 60–90 days. The flavor characteristics of SAB include a fruit and flower aroma, a sweet mouthfeel, and a long aftertaste (Jin et al., 2017; Xu et al., 2022).

Chinese traditional *Baijiu* is usually produced in an open environment using spontaneous solid-state fermentation technology. The microorganisms involved in SAB fermentation are derived from *Daqu* (the starter) and the workshop environment, and the functional microbes can use the nutrient substances in grains to multiply and produce flavor substances. Thus, the microbial composition and function during fermentation are crucial for the quality of SAB (Tan et al., 2019; Yuan et al., 2023). Like other traditional fermented foods, the production process of Chinese *Baijiu* generally involves grain saccharification and flavor fermentation according to microbial metabolic function: during the saccharification stage, starch is decomposed into reducing sugar by mold; then, the reducing sugar are converted into alcohol, esters and other flavor substances, including yeast and bacteria, by microbial metabolism (Jin et al., 2017; Shen et al., 2021). Thus, a comprehensive understanding of the microbial composition and function at different stages of *Baijiu* fermentation could increase our understanding of the performance and stability of the microbial community and the regulation of microbial metabolism of flavor components during grain fermentation.

Most traditional fermented food production involves a complicated multi-species microbial community, which usually forms spontaneously in a specific fermentation environment (Wu et al., 2021). Complex interactions among abiotic factors, microbes and food flavor could play a key role in microbial succession and flavor formation (Wang et al., 2021). Knowledge of environment-microbe-flavor interactions would be beneficial for understanding the production mechanism underlying food flavor in Chinese *Baijiu*. Flavor components can be driven by abiotic factors in fermentation environments, such as temperature (Wang et al., 2020), acidity (Ji et al., 2023), and amino acids (Wei et al., 2023). These abiotic factors also affect microbial succession at different stages of *Baijiu* fermentation. However, our understanding of the joint effects of abiotic and microbial factors on flavor during *Baijiu* fermentation is still poor, especially SAB. Thus, analyzing the relationships between abiotic and microbial factors and volatile components during SAB fermentation would be beneficial for managing the flavor profile and quality of SAB.

This study was aimed at revealing the effects of abiotic and microbial factors on the formation of flavor components during the entire SAB fermentation process. Recent research has mostly revealed microbial dynamics at the general level during the fermentation of Chinese *Baijiu*, hindering further exploration of specific functional microbial species changes and their contribution to the *Baijiu* flavor (Dong et al., 2022; Guan et al., 2023; Xu et al., 2023). Here, we investigated the microbial composition of fermented grains via the PacBio sequencing platform (third-generation sequencing technology), which can detect the microbial composition at the species level by obtaining the full-length sequences of the 16S rDNA and ITS regions (Wagner et al., 2016). The volatile components were measured by headspace solid-phase microextraction-gas chromatography–mass spectrometry (HS-SPME-GC–MS) detection method. Coupled with physicochemical property analysis, multivariate analysis was performed to reveal the abiotic and microbial effects on the production of flavor substances. This study provides a theoretical basis for understanding the role of the core microbial community and physicochemical factors in flavor formation during SAB fermentation.

## 2 Methods

### 2.1 Samples collecting

During SAB production, fermented grain samples were collected from a famous *Baijiu* factory located in Yibin, Sichuan Province, China, in 2022. SAB is fermented using five types of grains (sorghum, rice, glutinous rice, wheat and corn) as raw materials and *Daqu* as the starter in a pit cellar. *Daqu* is primarily made of wheat, and collects the fermentation microbes from the open environment by about 30 days of natural fermentation. The traditional solid-state fermentation process of SAB is as follows: fresh raw materials are crushed and mixed in proportion, and then approximately 80 °C water is added to moisten the grains (the water content reaches 70 %–80 %). After that, it was mixed with the fermented grains for steaming. The mixture was then cooled to 12–15 °C, stirred well with *Daqu* at a ratio of 28:100 (*Daqu*: fresh raw material), and then transferred to the pit cellar (3 m × 3 m × 5 m) for ethanol production and fermentation. *Daqu* used in this experiment was produced by the local *Baijiu* factory itself. According to the traditional fermentation technology of SAB, the entire fermentation process lasts for 88 days to achieve the sufficient enrichment of flavors (Liu et al., 2023). In this study, ten key fermentation points were chosen to collect fermented grain: fermentation days 0, 4, 7, 14, 21, 30, 50, 60, 70, and 88. A total of 250 g of fermented grain sample was collected from the middle layer (1.5 m) of each pit at each sampling time point, and the samples from three different pits were regarded as parallel samples. All the samples were stored at −20 °C for physicochemical analysis, DNA extraction and volatile substance detection.

### 2.2 Detection of physiochemical properties in fermented grains

The temperature of fermented grains was monitored in situ and recorded by the thermometer at each time point. About 5 g of fermented grain was dried at 105 °C for 3 h in an oven (Jinghong DHG9001, Shanghai, China), and then the weight loss of the sample after drying was used to evaluate the moisture content of the sample. The titratable acidity of the fermented grain was determined by titration with NaOH (0.1 M) to the endpoint of pH 8.2. The pH value of the fermented grain was obtained by detecting the suspension (10 g samples/ 100 mL) with a pH meter (Mettler Toledo, Swiss). The content of the reducing sugar in the samples was detected by Fehling's solution method (Cresswell, 1886). Residual starch content of the fermented grain was calculated by detecting the consumption of the reducing sugar after hydrolysis with HCl (20 %, *v*/v) for 30 min (Guan et al., 2020). All physicochemical tests of the fermented grains were determined in triplicate.

### 2.3 DNA extraction, 16S rDNA and ITS region full-length amplicon sequencing

Total genomic DNA of fermented grain was extracted by using the cetyltrimethyl ammonium bromide (CTAB) method (Kachiprath et al., 2018). After quantification, DNA was diluted to 1 ng/μL using sterile ultrapure water. The full-length of 16S rDNA region and ITS region were amplified by PacBio platform using 16S primers pair (forward primer: 5-AGAGTTTGATCCTGGCTCAG-3′, reverse primer: 5-GNTACCTTGTTACGACTT-3′) and ITS primers pair (forward primer: 5’-TACACACCGCCCGTCG-3′, reverse primer: 5-GCATATDANTAAGSGSAGG-3′), respectively. All PCR reactions were conducted in 30 μL volumes, containing 15 μL of Phusion® High-Fidelity PCR Master Mix with GC Buffer (New England Biolabs, Beijing, China), 0.2 mM of forward and reverse primers, and 10 ng template DNA. The PCR products were purified and used to construct SMRT Bell sequencing libraries after concentration adjustment. Full-length amplicon sequencing was performed on the PacBio platform of Allwegene Technology Co., Ltd. (Beijing, China).

### 2.4 Bioinformatics analysis

Raw data was filtered and removed primers, and then simple sequence repeat containing consecutive identical base numbers>8 was filtered out by CCS (SMRT Link v7.0). After that, Clean Reads of all fermented grain were clustered into OTUs (Operational Taxonomic Units) with 97 % identity by using Uparse software (Uparse v7.0.1001, http://drive5.com/uparse/, Edgar, 2013). And the representative sequences of OTUs as highest frequent sequence were annotated using SILVA database (http://www.arb-silva.de/) for bacterial species annotation and UNIT database (https://unite.ut.ee/) for fungal species annotation (Edgar, 2004; Quast et al., 2013). Raw sequencing data were uploaded to the Short Read Archive repository (SRA) of National Center for Biotechnology Information (NCBI, http://www.ncbi.nlm.nih.gov/Traces/sra/) under the BioProject accession number PRJNA740137.

### 2.5 Volatile substance analysis by HS-SPME-CS-MS

The volatile components in fermented grains were determined by HS-SPME-GC–MS conducted on an Agilent 7890B GC system coupled with an Agilent 5977B MSD (CA, USA). First, 10 g of each fermented grain sample was added to 25 mL of 1 % calcium chloride solution and soaked overnight at 4 °C. The suspension was ultrasonicated for 30 min and then centrifuged at 8000 ×*g* at 4 °C for 10 min. Seven mL of the supernatant was mixed with 14 mL of ultrapure water, 7 g of sodium chloride and 20 μL of internal standard (2-octanol, 100 mg/L). The samples were analyzed using the HS-SPME-GC–MS system with a 50/30 μm DVB-CAR-PDMS fiber (Supelco Co., Bellefonte, PA, USA) in splitless mode. The GC–MS running conditions were as follows: the temperature program of the oven started at 50 °C, increased to 220 °C at a rate of 3.0 °C/min, and was maintained for 5 min. Helium was used as the carrier gas at a constant flow rate of 1.0 mL/min. The electron ionization (EI) mass spectra mode was used with an ionization energy of 70 eV. The temperature of the ion source was 230 °C, and the mass range of the full scan mode was set from 20 to 500 amu. The retention indices (RIs) were calculated from the retention times of these compounds and n-alkanes (C7-C30) under the same GC–MS operating conditions. The compounds were identified by comparing the matching quality of mass spectral profiles ≥80 with those available in the National Institute of Standards and Technology (NIST 14) library (Gaithersburg, MD, USA) and further verified by comparing their retention indices calculated with those in the public data. The relative content of each compound was determined by comparing its peak area to the integrated peak area of the total compounds.

### 2.6 Statistical analyses

A principal coordinate analysis (PCoA) of the microbial communities was conducted using the “Vegan” package in R (version 4.2.1). Redundancy analysis (RDA) and Mantel tests were performed to reveal the effects of physicochemical properties on the microbial community. A heat map of volatile substances during fermentation was drawn using the “pheatmap” package. The principal component analysis (PCA) of volatile substances was analyzed using the “FactoMineR” package. The correlation networks between the key microbes and volatile substances were visualized with Cytoscape (version 3.7.1) according to Spearman's correlation analysis (|ρ| > 0.6 and *p* < 0.05). The potential functional profiles of the bacterial communities were predicted using PICRUSt2 (Langille et al., 2013), whereas the functional characteristics of the fungal OTUs were annotated using the FUNGuild database (Nguyen et al., 2016). Structural equation modeling (SEM) was performed to explore the effects of the physicochemical and microbial factors (diversity and function) on the composition of volatile metabolites (Wu et al., 2022).

## 3 Results

### 3.1 Changes of physiochemical properties during grain fermentation

**Fig. 1:**
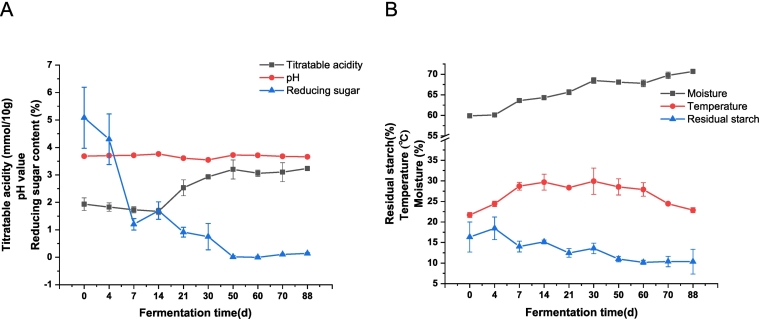
Dynamics of physicochemical properties in fermented grain of strong-aroma *Baijiu* (*n* = 3). A) titratable acidity, pH and reducing sugar. B) Moisture, temperature and residual starch.

The changes of temperature, moisture, acidity, pH, reducing sugar, and residual starch during the spontaneous fermentation of SAB are shown in Fig. 1. The initial temperature of the fermented grains was 21.7 °C, which increased significantly to 28.7 °C on the 7th day of fermentation. The temperature was then maintained between 28.3 °C and 29.9 °C for the next forty days and ultimately decreased to 22.8 °C until the end of grain fermentation. The moisture content of fermented grains gradually increased during grain fermentation and reached the highest level (74 %) on the 70th day. The pH remained stable during the first fourteen days of fermentation and then decreased to 3.54 after 30 days of fermentation. Finally, it increased to 3.65 and 3.7 until the end of grain fermentation. The titratable acidity of the fermented grains began to increase significantly to 2.56 after 21 days of fermentation and was maintained between 2.82 and 3.24. As the main substrate for microbial activity in grain, the reducing sugar content gradually decreased (5 % at the beginning of fermentation) and remained between 0.028 % and 0.144 % after 50 days of fermentation. Similarly, the residual starch content in fermented grains was 16–17 % at the early stage of fermentation, decreased to 10 % after 50 days of fermentation, and remained until the end of fermentation.

### 3.2 Microbial composition during the grain fermentation

**Fig. 2:**
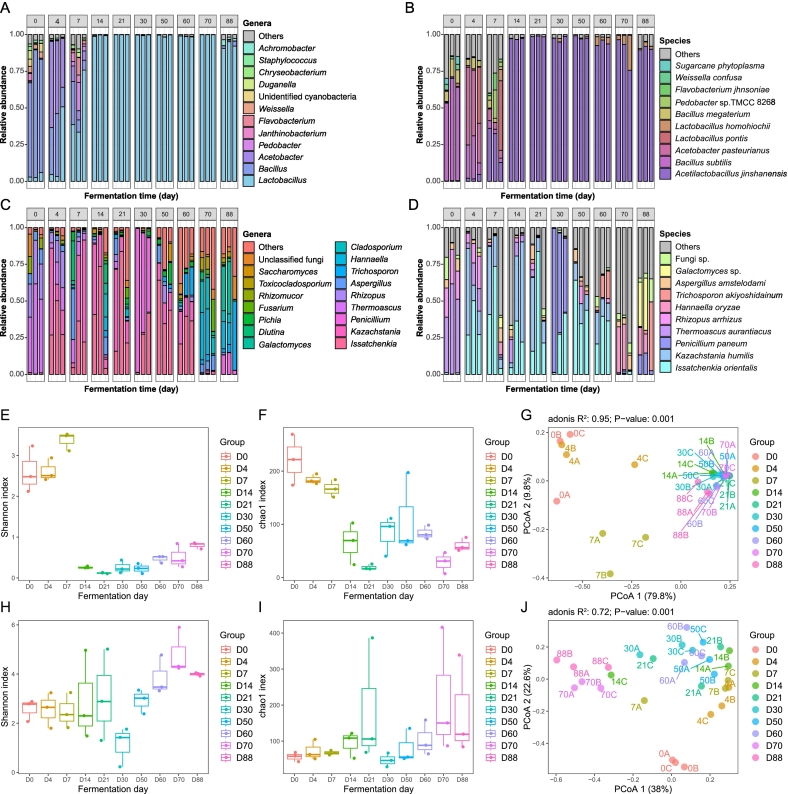
Microbial composition and diversity at different fermentation times of the strong-aroma *Baijiu* fermentation. Microbial abundance and succession shown at the genus level and the species level (A and B: bacteria, C and D: fungi). The genera that had an average abundance of >1 % and top 10 abundant species were shown in the chart. The non-abundant genera and species were grouped in the category named “others”. Shannon index and chao 1 index were shown (bacterial community: E and F; fungal community: H and I). Principal coordinates analysis (PCoA) of bacterial (G) and fungal (J) community based on the Bray-Curtis dissimilarity matrix.

To identify the key microbes involved in volatiles production, we analyzed the changes in the microbial community during SAB fermentation via full-length sequencing of 16S rRNA and the internal transcribed spacer (ITS) gene region. A total of 555,607 clean reads were obtained for the bacterial 16S rRNA region, and 291,522 clean reads were obtained for the fungal ITS gene region. For the bacterial community, the dominant bacteria in the fermented grains at different stages were *Lactobacillus*, *Bacillus*, *Staphylococcus*, *Pyroactinomyces* and *Weissiella*, which accounted for more than 91 % of the total relative abundance (Fig. 2A). At the species level, a total of 186 bacterial species were detected in the fermented grain at different stages. Among them, *Acetilactobacillus jinshanensis* (homotypic synonym: *L. jinshani*), *B. subtilis*, *Acetobacter pasteurianus*, *L. pontis*, *L. homohiochii*, and *B. megaterium* made up more than 1 % of the average relative abundance during the fermentation process (Fig. 2B). At the initial stage of grain fermentation (0–4 days), the composition of the bacterial community in fermented grains was dominated by *Bacillus*. However, as grain fermentation progressed, an anaerobic environment formed in the fermented pit, and *A. jinshanensis* gradually became the dominant bacteria, accounting for 92–99 % of the total bacteria during the later fermentation period.

For the fungal community, the dominant fungal genera during SAB fermentation were *Issatchenkia*, *Kazachstania*, *Thermoascus*, *Aspergillus*, *Rhizopus*, and *Lichtheimia*, accounting for 90.7 % of the total fungi. At the species level, a total of 478 fungal species were detected in the fermented grains (Fig. 2C). Among them, *Issatchenkia orientalis*, *Kazachstania humilis*, *Penicillium paneum*, *Thermoascus aurantiacus*, *Rhizopus arrhizus*, *Hannaella oryzae*, *Trichosporon akiyoshidainum*, *Aspergillus amstelodami*, *Galactomyces* sp., *Diutina rugosa*, and *Cladosporium cladosporioide* made up more than 1 % of the relative abundance during the fermentation process (Fig. 2D). In the initial stage of grain fermentation, *T. aurantiacus* and *R. arrhizus* dominated the fungal community, accounting for more than 50 % of the average relative abundance. However, as fermentation progressed, *I. orientalis* and *K. humilis* began to increase dramatically and became the dominant fungi during the middle and later stages.

### 3.3 Microbial diversity during the grain fermentation

Alpha rarefaction analysis (Fig. S1) revealed that most of the fungal and bacterial OTUs in the fermented grains were detected, and the sample size was sufficient to reveal microbial diversity across all the samples. Shannon and Chao1 alpha diversity indices revealed that the bacterial community diversity increased during the initial seven days of grain fermentation, and the diversity index decreased dramatically at fermentation day 21 and then maintained during the remaining fermentation time (Fig. 2E and F). Fungal community diversity slightly fluctuated before the twenty-first day of fermentation, dramatically decreased at the thirtieth day of fermentation, and then increased at the later stage of fermentation (Fig. 2H and I).

PCoA coupled with the Adonis test indicated that the composition of the microbial community significantly changed during grain fermentation (bacteria: R^2^ = 0.95, *p* = 0.001; fungi: R^2^ = 0.72, *p* = 0.001), and the PC1 and PC2 axes both accounted for 89.6 % of the total variance in the bacterial community (Fig. 2G) and 60.6 % of that in the fungal community (Fig. 2J). The bacterial composition significantly differed after the fourteenth day of fermentation, whereas the fungal community gradually changed during the SAB fermentation. In addition, the composition of the entire microbial community on fermentation days 0 to 7 was significantly different from that on fermentation days 14 to 88 (Fig. S2), indicating that the microbial community exhibited stage-specific variation in response to environmental changes in fermented grains. Thus, the whole SAB fermentation process can be divided into two stages according to microbial changes: stage 1 (fermentation days 0 to 14) and stage 2 (fermentation days 14 to 88).

### 3.4 Functional prediction of bacterial and fungal communities during the fermentation process

**Fig. 3:**
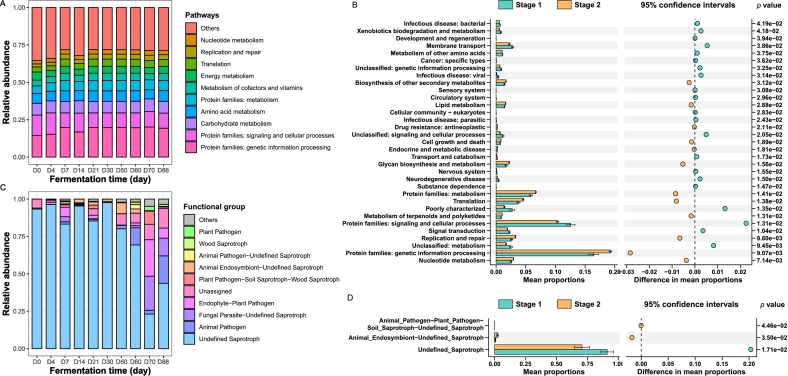
Functional profiles of microbial communities at different fermentation time of strong-aroma *Baijiu*. Predicted pathway of the bacterial community (A) and functional characteristics of the fungal community (C). Differences of microbial functional profiles (B: bacteria, D: fungi) between the different stages of fermentation were assessed using *t-*tests, and the significant differences indicated by *p* < 0.05.

To understand the microbial contributions to flavor metabolism and nutrient utilization involving the fermentation process, PICRUSt2 and FUNGuild were used to predict the functions of the bacterial and fungal communities, respectively (Fig. 3). In the bacterial community, pathways related to protein, amino acids, carbohydrates, cofactors, vitamins, and energy metabolism were enriched during fermentation. Fungal functions were determined by classifying the fungal OTUs into various nutritional categories, and the fungi identified were enriched in genes associated with saprotroph, animal and plant pathogens. The difference analysis of the microbial functional profiles further revealed that a total of 33 pathways were significantly different during SAB fermentation. Among them, the expression of bacterial pathways involved in protein, lipid and secondary metabolites was greater during the middle and later stages (Stage 2) of grain fermentation, which indicated that the flavor substances produced by the amino acids accumulated in the later stage. In addition, the relative abundance of unidentified saprotrophs was high in the early stage (stage 1) of grain fermentation, whereas the relative abundance of animal endosymbiont-unidentified saprotrophs significantly increased in the middle and late stages (stage 2) of fermentation. These results indicated that microbial function profiles significantly changed during fermentation.

### 3.5 Physicochemical factors driving microbial succession during the grain fermentation

**Fig. 4:**
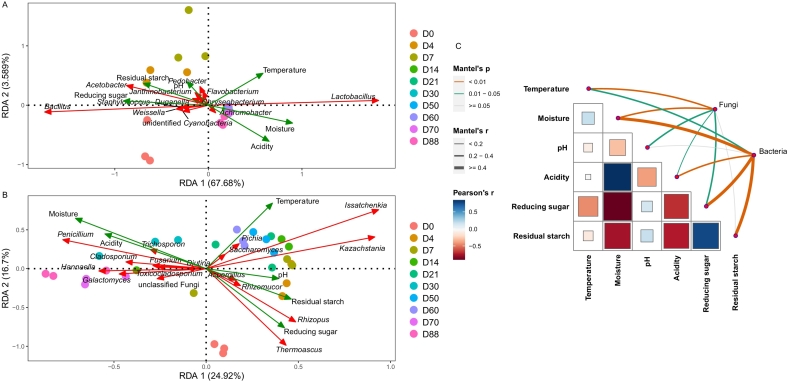
Relationships between dominant microbes and physicochemical properties during the strong-aroma *Baijiu* fermentation. Redundancy analysis (RDA) on bacterial (A) and fungal (B) microbiota with physicochemical properties in fermented grains of strong-aroma *Baijiu*. Microbes and physicochemical properties were represented by red and blue lines with arrow. Colorful dots represents the samples in different brewing stages. Mantel tests related with microbial community and physicochemical properties (C), and edge colour denoted the statistical significance based on 999 permutations. (For interpretation of the references to colour in this figure legend, the reader is referred to the web version of this article.)

To reveal the key driving factors for microbial succession, we analyzed the correlations between physicochemical factors and the microbial community in the fermented grain (Fig. 4). Mantel tests revealed that temperature (bacteria: *r* = 0.226, *p* = 0.02; fungi: *r* = 0.308, *p* = 0.001), moisture (bacteria: *r* = 0.659, *p* = 0.01; fungi: *r* = 0.369, *p* = 0.01), acidity (bacteria: *r* = 0.254, *p* = 0.001; fungi: *r* = 0.153, *p* = 0.011) and reducing sugar (bacteria: *r* = 0.81, *p* = 0.001; fungi: *r* = 0.222, *p* = 0.016) were significantly related to the bacterial and fungal communities. RDA was also performed to identify the effects of physicochemical factors on the variation in microbial species. For the bacterial community, RDA 1 and RDA 2 contributed 68.89 % and 4.785 % of the variation in the bacterial community, respectively. Temperature, moisture, and acidity were positively associated with *Lactobacillus,* whereas pH, residual starch and reducing sugar were positively associated with *Bacillus*, *Acetobacter*, *Weissella* and other genera. For the fungal community, RDA 1 and RDA 2 contributed 25.85 % and 15.82 % of the variation in the fungal community, respectively. Temperature, pH, residual starch and reducing sugar were positively related to *Issatchenkia*, *Kazachstania*, *Rhizopus* and *Thermoascus,* whereas moisture and acidity were positively related to *Penicillium*, *Trichosporon*, *Galactomyces* and *Cladosporium.*

### 3.6 Volatile components during grain fermentation

**Fig. 5:**
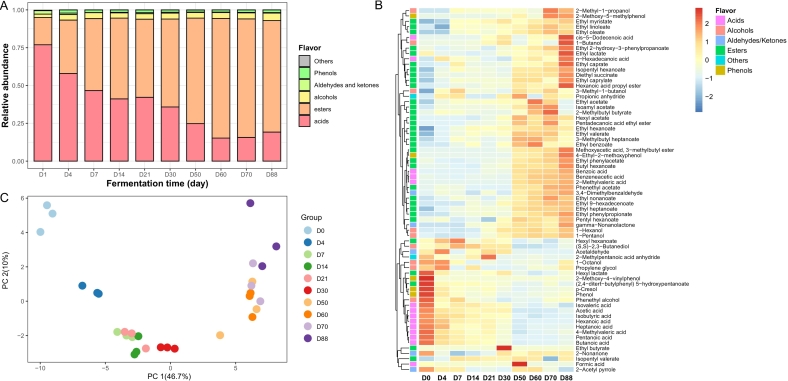
Changes of volatile substance during the strong-aroma *Baijiu* fermentation. (A) Proportions of main flavor components in the fermented grains of SAB fermentation (*n* = 3). (B) Heatmap showing the frequency distribution of volatile components in fermented grains of strong-aroma *Baijiu*. (C) Principal component analysis (PCA) of volatile components at different fermentation time. A total of 68 aroma components were presented in the chart. The different colour intensities indicated the standardizing value of aroma components content. The clustering along y axis was based on relative contents of volatile components present in each sample.

A total of 68 volatile components, including 33 esters, 14 acids, 9 alcohols, 5 phenols, 2 aldehydes, 2 ketones and three other flavor components, were identified from the fermented grains via HS-SPME-GC–MS (Fig. 5, Table S1). Among them, acetic acid, caproic acid, isovaleric acid and n-butyric acid were abundant in fermented grains, and hexanol, butanol, octanol, phenyl ethanol, and 3-methyl-1-butanol were identified as the main alcohols. Moreover, the main esters with high volatile contents during fermentation were ethyl caproate, hexyl caproate, ethyl lactate, bornyl acetate, isoamyl acetate, ethyl caprylate, ethyl linoleate, ethyl phenylacetate, and more. As the fermentation process progresses, the total content of volatile components, especially ester substances, in fermented grains gradually increased. The esters accounted for only 17.9 % of the total volatile components on day 0 of fermentation and gradually increased with fermentation, accounting for 73.4 %–79.1 % of the total volatile components at the later stage of fermentation.

PCA revealed that the volatile components were clustered into two groups throughout the fermentation process, and the volatile components in fermented grain before 30 days of fermentation (D0 to D30) were significantly separated from those after 30 days of fermentation (D50 to D88) along the PCA 1 axis. A heat map further revealed that acetaldehyde, ethyl butyrate, isobutyric acid, acetic acid, and (*S*,*S*)-2,3-butanediol were the main compounds present in the fermented grains before fermentation for 30 days. After that, the contents of most volatile components, especially esters, acids and alcohols, gradually increased at the middle and later stages of fermentation. In general, the volatile components in fermented grains distinctly varied with the SAB fermentation process.

### 3.7 Correlation of microbial species and volatile components during the fermentation process

**Fig. 6:**
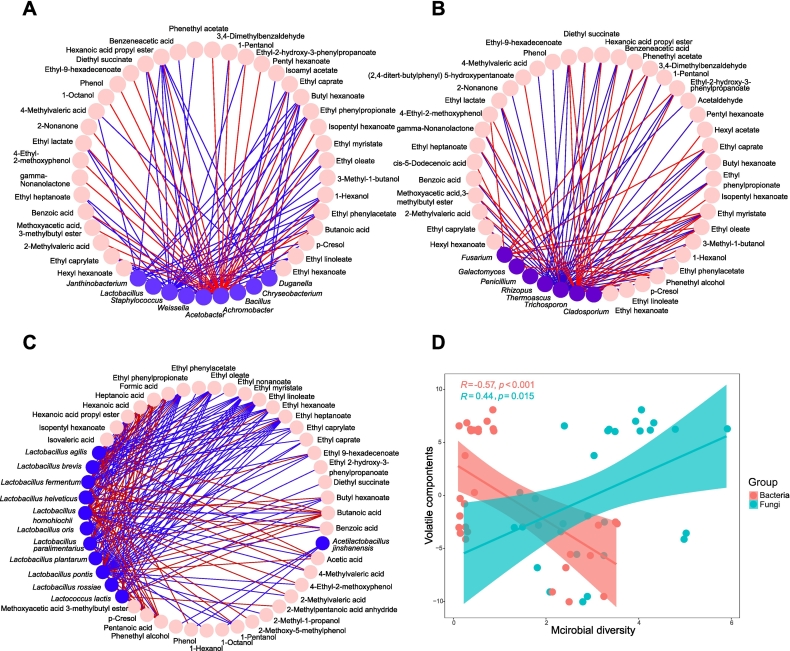
Correlation network analysis between bacterial genera (A), fungal genera (B), *Lactobacillus* species (C) and aroma compounds during the strong-aroma *Baijiu* fermentation. Significant spearman's correlations (|ρ| > 0.6, *p* < 0.05) were shown in diagram. Microbes and volatile substances were represented by blue and pink modules respectively, and red and blue lines refer to the positive and negative correlations between dominant genera and aroma compounds respectively. The relationship between volatile substances and microbial shannon diversity index was evaluated with pearson correlation. The composition of volatile substances were represented by the first axis of the PCA. Colorful area denoted 95 % confidence intervals. (For interpretation of the references to colour in this figure legend, the reader is referred to the web version of this article.)

Here, Shannon diversity index (SDI) was used to reveal the relationships between microbial diversity and volatile components, and the results revealed that fungal (*R* = 0.44, *p* = 0.015) and bacterial diversity (R = -0.57, *p* < 0.001) were significantly related to the contents of volatile components (Fig. 6). The fungal and bacterial communities play key roles in volatile metabolism. Moreover, the bacterial community rather than the fungal community might improve the production of key volatile components during the fermentation of SAB because of its relatively high coefficient and significance. Furthermore, the relationships between major microbial genera, *Lactobacillus* species and volatile components were visualized using Cytoscape. And correlation networks further confirmed that these microorganisms contributed greatly to the accumulation of flavor components. Among the fungal genera, *Fusarium*, *Cladosporium*, *Trichosporon* and *Penicillium* were positively related with most of volatile components, while *Rhizopus* and *Thermoascus* had a significant negative correlation with the esters and acids. The bacterial genera, *Acetobacter*, *Janthinobacterium*, *Weissella*, and *Staphylococcus* were negatively correlated with the the volatile components except *Achromobacter*, positively with the esters, alcohols and acids. Among *Lactobacillus* species, *L. agilis*, *L. brevis*, *L. fermentum*, *L. helveticus*, *L.homohiochii*, *L. paralimentarius*, *L. plantarum*, *L. pontis*, *L. rossiae*, and *L. lactis* were significantly correlated with most of the volatile component, especially positively related with acids.

### 3.8 Relationships among physicochemical properties, microbial factors and volatile metabolites

**Fig. 7:**
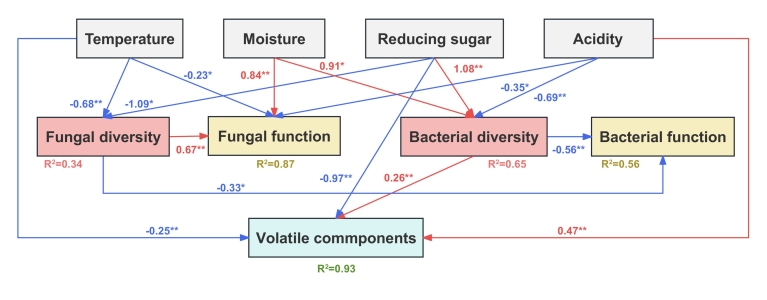
Structural equation model showing the effect of abiotic (temperature, moisture, reducing sugar and acidity) and microbial factors (diversity and function of fungal and bacterial community) on volatile metabolites. Significant relationships are shown in the chart with solid line. Red line indicates positive relationship and blue indicates negative relationship. R^2^ represents the total percentage of variances explained for each response variable. (For interpretation of the references to colour in this figure legend, the reader is referred to the web version of this article.)

Here, structural equation modeling (SEM) was used to reveal the effects of physicochemical and microbial factors on volatile components, and the significant relationships among physicochemical factors (temperature, moisture, reducing sugar and acidity), microbial factors (diversity and function of fungi and bacteria), and the compositions of volatile components are shown in Fig. 7. The SEM revealed that bacterial diversity (*b* = 0.26, *p* < 0.01) and acidity (*b* = 0.47, *p* < 0.01) had significantly positive relationships with volatile components, whereas temperature (*b* = −0.25, *p* < 0.01) and reducing sugar (*b* = −0.97, *p* < 0.01) had negative effects on volatile components. However, fungal diversity and function were not significantly correlated with volatile components. The R^2^ value of SEM revealed that bacterial diversity explained 96 % of the variation in volatile components coupled with three physicochemical factors. In addition, temperature, moisture, reducing sugar and acidity had significant effects (*p* < 0.01) on fungal diversity and function, and these physicochemical factors explained 34 % and 87 % of the variation in fungal diversity and function, respectively. Moisture, reducing sugar and acidity could regulate bacterial diversity, explaining 65 % of the variation in bacterial diversity. In brief, bacterial community, acidity, temperature and reducing sugar were the key factors regulating the production of volatile components during SAB fermentation.

## 4 Discussions

In traditional fermentation of Chinese *Baijiu*, its flavors quality relies largely on microbial metabolic activity and abiotic factors in fermented grain (Wu et al., 2021). In this study, we focused on SAB fermentation and explored the abiotic and microbial contributions for volatile components during the entire *Baijiu* fermentation process.

During SAB fermentation, the microorganisms in fermented grains gradually formed a stable and simplified community in response to environmental pressure changes caused by microbial activities. Temperature, moisture, reducing sugar and acidity were identified as the major abiotic factors driving the temporal variations in microbes during different fermentation stages. Temperature, moisture and reducing sugar are essential factors for microbial growth and metabolism and can significantly affect fungal and bacterial composition during fermentation (Guan et al., 2020). Acid in fermented grains reportedly plays a crucial role in microbial metabolism, and moderate acid could improve the production of flavor substances (Ji et al., 2023). Lactic acid bacteria (LAB) constitute the dominant bacterial taxon in most traditional fermented foods, and they can decrease the pH value of fermented grain by secreting lactic acid and acetic acid, which are the key precursors of ethyl lactate and ethyl acetate in *Baijiu* fermentation (Pang et al., 2021). As the dominant bacterial species, *Acetilactobacillus jinshanensis* is an important indicator of microbial succession, and it increased dramatically and became the absolute dominant bacterial species after the 14th day of SAB fermentation. *A. jinshanensis* was first identified from vinegar fermentation cultures in China (Yu et al., 2020). This species has strong acid resistance and can improve the flavor of fermented food by producing volatile metabolites (acids, alcohols and esters) based on transcriptomic analysis (Liao et al., 2023). And *A. jinshanensis* was also reported to be the dominant species, accounting for more than 50 % of the bacterial community involved in traditional Chinese *Baijiu* fermentation (Liu et al., 2023). Interestingly, *A. jinshanensis* unexpectedly had few relationships with the flavor components in our study, although it accounted for most of the relative bacterial abundance during fermentation. In addition, functional analysis revealed that *A. jinshanensis* was significantly associated with pathways related to amino acid, glycan and secondary metabolite metabolism (Table S2), indicating its crucial role in the production of flavor esters and alcohol during SAB fermentation. And this lactic acid bacteria abundance was also significantly correlated with the flavor-producing yeasts in fermented grains. This phenomenon revealed that *A. jinshanensis* may be more likely to improve the biosynthesis of key flavor components by microbial synergistic effects rather than producing flavor profiles as the key taxa (Du et al., 2020; Yuan et al., 2023). Overall, *A. jinshanensis* can increase the production of flavor esters and alcohols by regulating the microbial community during the SAB fermentation (Chen et al., 2024; Xu et al., 2022).

Compared with the bacterial community, the fungal community tended to gradually change in response to changes in the fermentation environment. During pit fermentation, the increase in pH resulting from lactic acid produced by LAB might inhibit the proliferation of unnecessary microorganisms that are harmful to flavor metabolism. *Lactobacillus* provides a convenient environment for the growth of acid-resistant functional yeast (Chen et al., 2022; Liu et al., 2023), which is consistent with the significant relationships among pH, acidity and the fungal community. As common yeasts in food fermentation, *I. orientalis* and *K. humilis* became the dominant species after 30 days of SAB fermentation. *I. orientalis,* with its high temperature and acid resistance, can grow in low-pH fermented grains in pit cellar, and produce a great deal of ethanol and higher alcohols, which gives SAB a sweet taste (Guan et al., 2023; Jiao et al., 2022). In addition, *I. orientalis* was significantly positively related to *K. humilis* and *S. cerevisiae* (*p* *<* 0.01, Table S3), which was consistent with the findings of a previous study. *I. orientalis* might collaborate with *Saccharomyces* and *Kazachstania* to improve the production of ethyl caproate and alcohols at the middle and last stages of SAB fermentation (Dong et al., 2024). *Kazachstania*, another dominant genus in the early and middle stages of fermentation, has been reported to contribute greatly to flavor components (such as alcohols) during SAB fermentation (Ling et al., 2021). In addition, *Kazachstania* can also inhibit lactic acid bacteria from producing lactic acid, which interact with lactic acid bacteria to maintain a stable fermentation environment for flavor component production (Wang et al., 2018).

Previous investigations have addressed microbial succession in different fermentation stages of Chinese *Baijiu* according to the brewing process or fermentation parameters (Shen et al., 2021; Lin et al., 2022). However, microbial changes are sometimes not consistent with the division of fermentation stages (Lin et al., 2022). Moreover, all stages of SAB fermentation occur in only one pit cellar, which has no clear division of fermentation stages during industrial production. Thus, research on fermentation mechanisms based on microbial dynamics is beneficial for understanding microbial succession and flavor formation. Here, microbial succession involved composition and function driven by abiotic factors in fermented grains, and it can be divided into two stages. During the first stage of SAB fermentation (D0 to D14), the bacterial community first rapidly changed, and lactic acid bacteria became the dominant microbial group, which produced large amounts of lactic acid to inhibit unwanted microbial groups. Moreover, the core microbes degraded macromolecular nutrients in grains into reducing sugar, amino acids and other small molecular substances. During the second stage (D14-D88), flavor-producing yeast with high acid tolerance became the functional dominant group in fermented grain and uses alcohols and acids to produce a large number of flavor esters.

Flavor characteristics of Chinese *Baijiu* are determined mainly by microbial metabolism during the grain fermentation process, and microbial dynamics result mainly from temporal variations in environmental factors (Zou et al., 2018). During the SAB fermentation process, the flavor components in fermented grain significantly changed, similar to the microbial community, and they significantly differed from those observed after 30 days. Microbial changes involving species composition and functional profiles took place earlier, with significant changes after 15 days of fermentation, which might drive the production of flavor components in fermented grains. The SEM results also supported this view and highlighted that the bacterial community could improve the production of volatile components in fermented grain. *Lactobacillus,* the dominant bacterial genus in most Chinese *Baijiu* fermentation, is involved in the biosynthesis of various flavor compounds, such as esters, alcohols and acids (Du et al., 2020; Huang et al., 2020; Xu et al., 2023). In this study, the abundance of several *Lactobacillus* species were significantly correlated with the abundance of volatile components, although that of *A. jinshanensis* was also correlated with the levels of a few volatile components. Among these species, *L. homohiochii* was significantly positively correlated with the contents of main esters, alcohols and acids, which indicated that low-abundance bacteria might contribute more strongly to the production of the main flavor substances than dominant bacteria during SAB fermentation. Although SEM revealed that fungal diversity and function were not significantly related to the content of volatile components, the regression analysis and Mantel tests revealed that the fungal diversity (*R* = 0.44, *p* = 0.015) and functional profile (*R* = 0.41, *p* *<* 0.001) were significantly related to volatile components, indicating that the fungal community still plays an important role in driving the production of flavor substances (Liu et al., 2021). The correlation analysis revealed that *Rhizopus*, *Thermoascus*, *Trichosporon*, and *Fusarium* were significantly related to most volatile components. These dominant genera might improve the production and accumulation of esters, alcohols and acids in fermented grains (Zhang et al., 2020).

Most previous studies have focused on the effects of microorganisms on volatile components during grain fermentation, but the relationships between physicochemical properties and volatile components have often been ignored (Dong et al., 2022; Luo et al., 2023). Here, we explored the effects of abiotic factors on volatile components. Temperature, acidity and moisture were identified as the key abiotic factors affecting the volatile components during SAB brewing according to a structural equation model, which accounted for 96 % of the variation in volatile components coupled with bacterial diversity. Unlike in previous studies, abiotic factors had a direct influence rather than an indirect influence on volatile components, and they might provide substrate or form a suitable environment for the production of flavor components. Overall, our study revealed that abiotic factors drive fungal and bacterial community succession, both of which affect the composition of flavor substances. This work provides a potential way to regulate the flavor and quality of SAB.

## 5 Conclusion

In summary, we systematically explored how the microbial community and physicochemical factors influence the production of flavor components during the SAB brewing. In the entire fermentation process, the microbial composition and functional profiles changed rapidly in response to physicochemical factors (temperature, moisture, acid and reducing sugar). And the changes in microbial community and physicochemical factors jointly drove the flavor production in fermented grain. Our results further revealed that the bacterial community was a crucial regulator in improving the flavor profiles coupled with temperature, acidity and reducing sugar compared to fungal community. This study provides insight for improving *Baijiu* quality and food industry modernization by regulating the bacterial community and key abiotic factors.

## CRediT authorship contribution statement

**Bin Lin:** Writing – original draft, Visualization, Methodology, Investigation, Formal analysis. **Jie Tang:** Investigation, Formal analysis, Data curation. **Qun Li:** Resources, Investigation. **Liping Zhu:** Visualization, Resources. **Wei Jiang:** Formal analysis, Data curation. **Hanbing Ke:** Resources. **Zhang Wen:** Resources, Formal analysis. **Huaichen Liu:** Supervision, Resources. **Shengzhi Yang:** Supervision, Funding acquisition. **Qiang Yang:** Supervision, Funding acquisition. **Shenxi Chen:** Writing – review & editing, Writing – original draft, Supervision, Conceptualization. **Peijie Han:** Writing – review & editing, Supervision, Funding acquisition.

## Declaration of competing interest

The authors declare that they have no known competing financial interests or personal relationships that could have appeared to influence the work reported in this paper.

## Data Availability

Raw sequencing data were uploaded to the Short Read Archive repository (SRA) of National Center for Biotechnology Information (NCBI, http://www.ncbi.nlm.nih.gov/Traces/sra/) under the BioProject accession number PRJNA740137.

## Appendix A Supplementary data

Supplementary material 1 **Fig. S1.** Rarefaction analysis of microbial community in different fermentation time of strong-aroma *Baijiu*. (A) Fungi; (B) Bacteria. Supplementary material 1

Supplementary material 2 **Fig. S2.** Constrained principal coordinates analysis (PCoA) of the whole microbial community based on the Bray-Curtis dissimilarity matrix. Supplementary material 2

Supplementary material 3 Image 1
