# Supplementary figures and images for: Bacterial community improves the volatile components coupled with abiotic factors during the spontaneous fermentation of Chinese strong-aroma Baijiu
Source: Food Chem X. 2024 Dec 9;25:102068. doi: 10.1016/j.fochx.2024.102068 (PMC11699094; doi:10.1016/j.fochx.2024.102068)

A

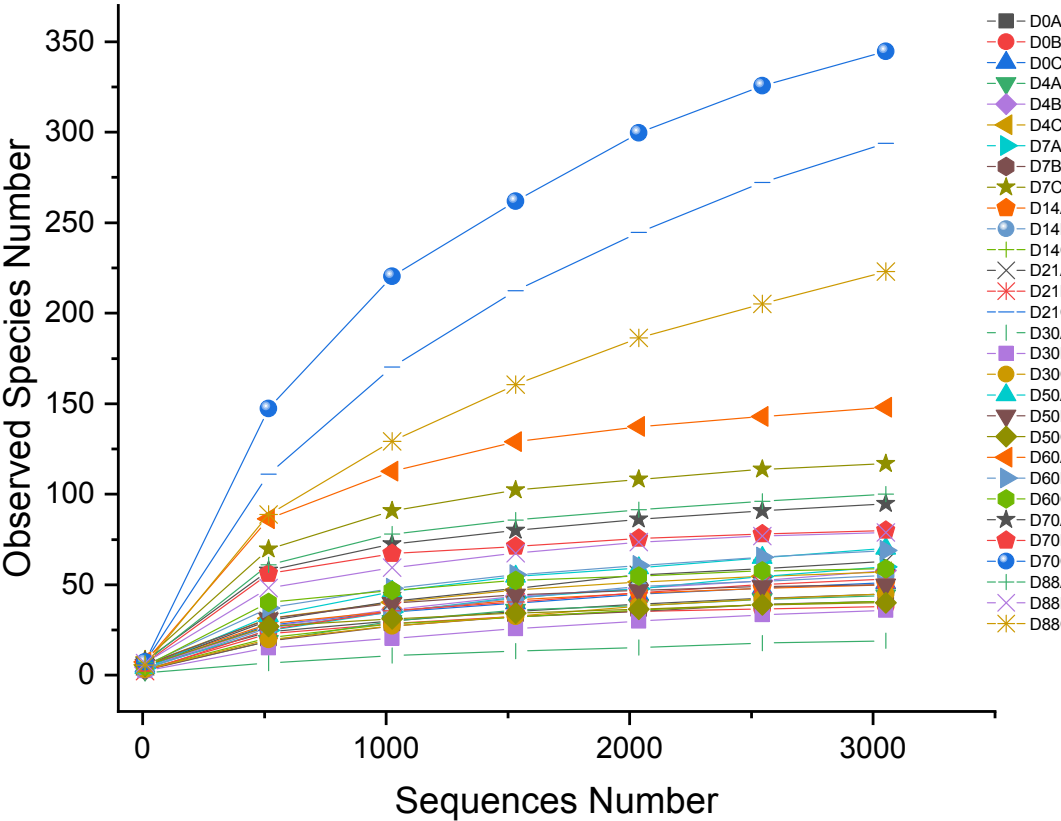

B

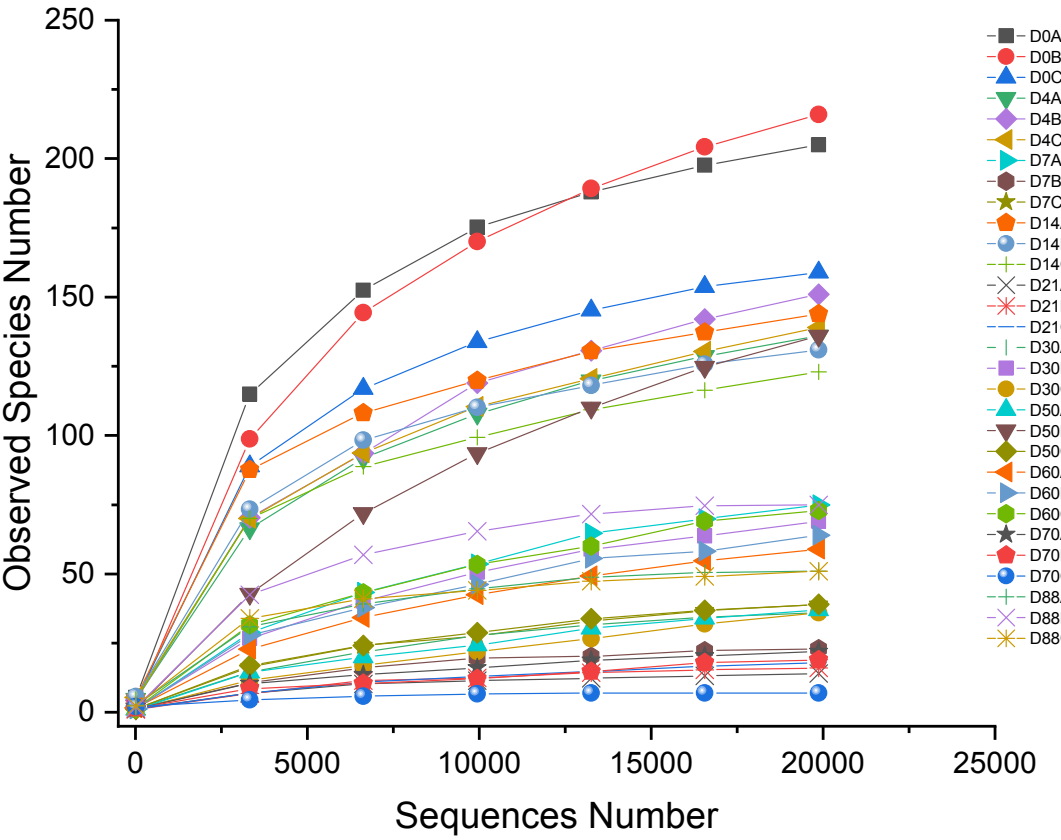

Supplement: Supplementary material 1 — Fig. S1. Rarefaction analysis of microbial community in different fermentation time of strong-aroma Baijiu. (A) Fungi; (B) Bacteria. [file mmc1.pdf]

adonis R<sup>2</sup>: 0.82; P-value: 0.001

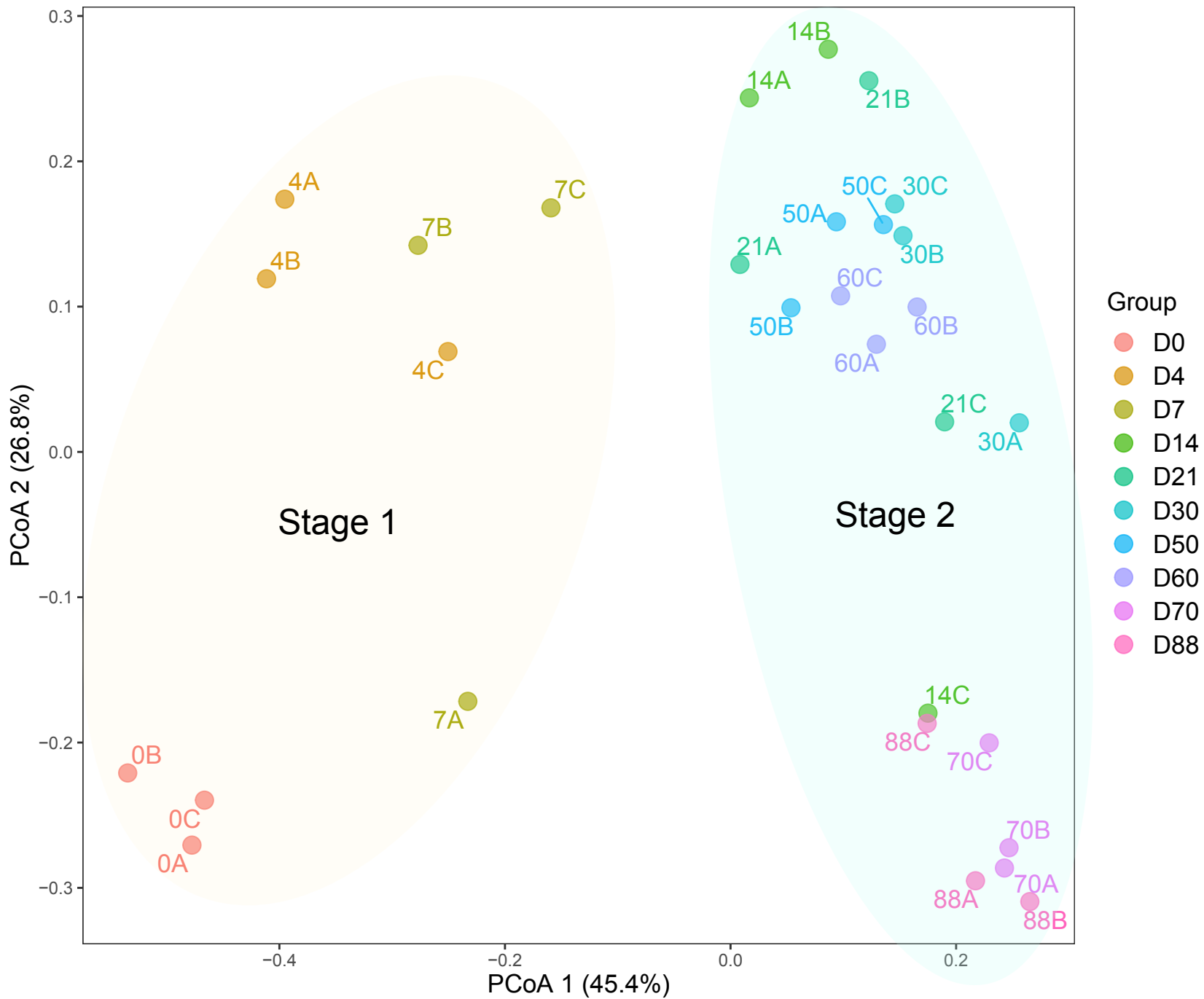

Supplement: Supplementary material 2 — Fig. S2. Constrained principal coordinates analysis (PCoA) of the whole microbial community based on the Bray-Curtis dissimilarity matrix. [file mmc2.pdf]
